# Watch brain circulation in unexplained progressive myelopathy: a review of Cognard type V arterio-venous fistulas

**DOI:** 10.1007/s10072-023-06870-1

**Published:** 2023-06-29

**Authors:** Amedeo De Grado, Chiara Manfredi, Agostino Brugnera, Elisabetta Groppo, Luca Valvassori, Federica Cencini, Alessandra Erbetta, Elisa Ciceri, Rosanna Lerario, Alberto Priori, Emma Scelzo

**Affiliations:** 1 Clinica Neurologica, Polo Universitario San Paolo, ASST Santi Paolo e Carlo, Dipartimento di Scienze della Salute, Università degli Studi di Milano, Via Antonio Di Rudinì, 8, Milan, Italy; 2 Department of Human and Social Sciences, University of Bergamo, Bergamo, Italy; 3 Department of Neuroradiology, ASST Santi Paolo E Carlo, Milan, Italy; 4 Department of Neuroradiology, Fondazione IRCCS Istituto Neurologico Carlo Besta, Milan, Italy; 5 Department of Imaging Radiology and Interventional Neuroradiology, Fondazione IRCCS Istituto Neurologico Carlo Besta, Milan, Italy; 6 Institute of Bari, Spinal Unit, ICS MAUGERI SPA SB, IRCCS, Bari, Italy; 7 Aldo Ravelli” Center for Neurotechnology and Experimental Brain Therapeutics, University of Milan, Milan, Italy

**Keywords:** Intracranial dural arterio-venous fistulas (iDAVFs), Intracranial vascular malformations, Myelopathy, Spinal cord disease

## Abstract

**Background:**

Intracranial dural arterio-venous fistulas are pathological anastomoses between arteries and veins located within dural sheets and whose clinical manifestations depend on location and hemodynamic features. They can sometimes display perimedullary venous drainage (Cognard type V fistulas—CVFs) and present as a progressive myelopathy. Our review aims at describing CVFs’ variety of clinical presentation, investigating a possible association between diagnostic delay and outcome and assessing whether there is a correlation between clinical and/or radiological signs and clinical outcomes.

**Methods:**

We conducted a systematic search on Pubmed, looking for articles describing patients with CVFs complicated with myelopathy.

**Results:**

A total of 72 articles for an overall of 100 patients were selected. The mean age was 56.20 ± 14.07, 72% of patients were man, and 58% received an initial misdiagnosis. CVFs showed a progressive onset in 65% of cases, beginning with motor symptoms in 79% of cases. As for the MRI, 81% presented spinal flow voids. The median time from symptoms’ onset to diagnosis was 5 months with longer delays for patients experiencing worse outcomes. Finally, 67.1% of patients showed poor outcomes while the remaining 32.9% obtained a partial-to-full recovery.

**Conclusions:**

We confirmed CVFs’ broad clinical spectrum of presentation and found that the outcome is not associated with the severity of the clinical picture at onset, but it has a negative correlation with the length of diagnostic delay. We furthermore underlined the importance of cervico-dorsal perimedullary T1/T2 flow voids as a reliable MRI parameter to orient the diagnosis and distinguish CVFs from most of their mimics.

## Introduction

Intracranial dural arterio-venous fistulas (iDAVFs) are rare malformations characterized by pathological anastomoses connecting arterial dural branches and dural sinuses, mostly the cavernous sinus and/or cortical veins. Arterial branches may arise from the external and internal carotid arteries and/or from the vertebrobasilar system. IDAVFs account for 10–15% of intracranial vascular malformations [1], representing about 6% of all supratentorial and 35% of all infratentorial vascular malformations [2]. They are generally acquired and associated with several predisposing factors such as history of craniotomy, head trauma, previous dural sinus infection or thrombosis, and genetic thrombosis predisposing mutations (heterozygous factor V Leiden and protein C/S deficiency) [1]. The mean age of diagnosis is between the fifth and the sixth decades, with a male-to-female ratio of 1.

Clinically, iDAVFs can present with both hemorrhagic and non-hemorrhagic symptoms, depending on the grade and anatomical localization. Hemorrhagic symptoms are typically characterized by lobar (or sublobar) hemorrhages, venous infarctions, and subdural hematomas; non-hemorrhagic symptoms are extremely variable, ranging from non-localizing signs such as intracranial hypertension with papilledema, headache, and nausea/vomiting to focal signs like seizures, cranial neuropathies, and pulsatile tinnitus. Chronic complications such as glaucoma, hydrocephalus, dementia, parkinsonism, and slowly progressive myelopathy are also possible [1].

IDAVFs are usually classified according to Borden-Shucart’s [3] or Cognard’s classifications [4], both strictly related to prognosis: the higher the grade, the worse the prognosis (see Table 1).

**Table 1:** Cognard classification

Cognard classification	Features
I	Drains into a dural sinus with anterograde flow
II	Drains with retrograde flow either into a sinus (IIA) or into cortical veins (IIB)
II A + B	Drains with retrograde flow into a sinus and into cortical veins
III	Drains into cortical veins without venous ectasia
IV	Drains into cortical veins with venous ectasia
V	Drains into spinal venous system

Cognard type V fistulas (CVFs) display perimedullary venous drainage and are associated with myelopathy in 50% of cases [4, 5]. These entities are extremely rare; until 2016, only 54 cases of CVFs had been described [2]. In 2020 Hou et al. reported 73 patients with CVFs, 57 of which presented with either paraparesis or tetraparesis [6]. CVFs’ clinical presentation is highly variable, but the classical picture is that of a middle-aged man with ascending tetraparesis (62%), sphincter dysfunction (34%), bulbar symptoms (31%), and a sensory level typically developing over several months; nevertheless, up to 50% of cases can present with acute onset [7]. Small vessel thrombosis, infarct or hemorrhage, are believed to be responsible for rapid worsening or acute onsets [2].

The pathophysiology of myelopathy and brainstem engorgement is similar to that described for type I–IV fistulas, involving congestion and dilation of the venous system, but with the involvement of perimedullary veins instead of cortical veins [8]. However, as noted by Brunereau and colleagues, not all CVFs cause myelopathy [9]. Some authors speculate that in a subset of patients, a medullary-radicular vein connecting the spinal perimedullary venous network to the epidural venous system at the cervical level may prevent the establishment of spinal cord venous hypertension, while the absence of the communicating vein may predispose to engorgement of cervico-thoracic perimedullary veins, leading to medullary edema and rarely spinal infarct due to poor arterial supply [9]. Two other possible theories to explain spinal cord involvement in CVFs are arterial steal and direct compression of the spinal cord by enlarged veins, clot, or varicose vessels [2].

Due to their rarity, these entities are seldom suspected, resulting in a diagnostic delay up to many years (average time 220–343 days) [10]. Whether this delay affects patients’ life expectancy and the grade of residual disability is still a matter of debate. It is noteworthy that El Asri et al. postulated the absence of correlation between diagnostic delay and the clinical outcome in patients with paraparesis, quadriparesis, or bulbar dysfunction. They also found that 38% patients with CVFs died or did not improve significantly despite the treatment, whereas 26% of patients showed an improvement after the treatment but still had a moderate disability, highlighting that the outcome of CVFs can often be life changing. Nevertheless, treatment resulted in complete recovery or noticeable improvement (defined as persistence of only mild symptoms) in 36% of cases [10].

The objective of this article is to review the literature describing the clinical and radiological spectrum of CVF presentation, starting with a representative case, and to investigate whether there are any reliable clinical or radiologic parameters that could help clinicians in suspecting the diagnosis. The possibility of a misdiagnosis due to many “iDAVFs mimics” is another key point of our study; CVFs may cause clinical and radiological findings very similar to a variety of inflammatory, infectious, or vascular diseases (i.e., spinal dural fistulas) affecting the spinal cord. This virtual absence of pathognomonic signs makes the diagnosis extremely challenging and currently possible only in a few specialized centers.

Furthermore, we aim to assess whether specific clinical and radiological findings impact diagnostic delay and prognosis, and if there is a correlation between clinical and/or radiological signs and clinical outcomes.

## A representative case

**Fig. 1:**
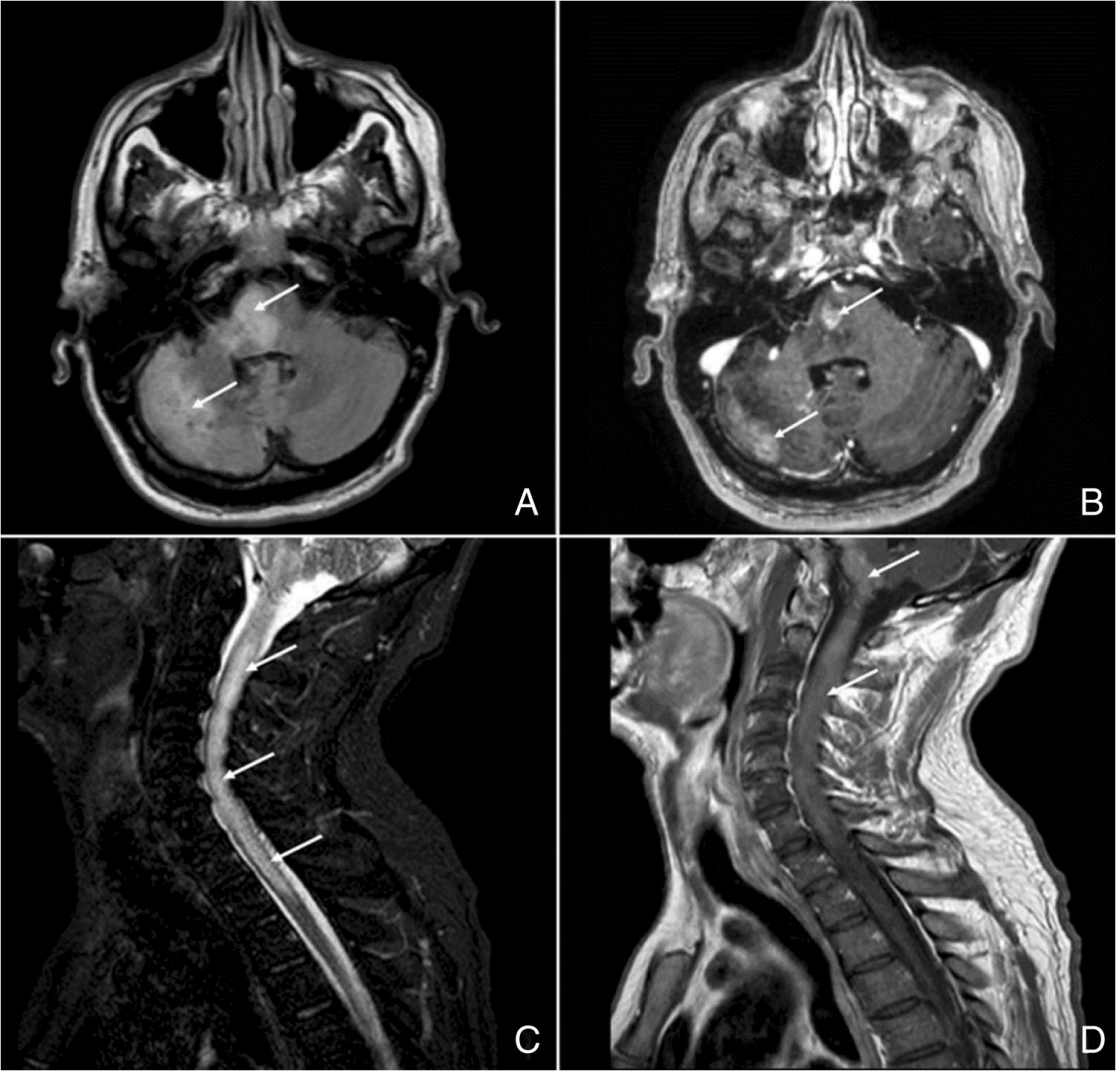
MRI at the diagnosis.** A** Axial FLAIR sequence shows hyperintensities in the pons and in the right cerebellar hemisphere (white arrows). **B** Axial T1-weight image obtained after gadolinium administration reveals enhancement of the same areas (white arrows). **C** Sagittal T2-weighted image shows intramedullary hyperintensity from the medulla oblongata to D2 vertebra level, with swollen cervical spinal cord (white arrows). **D** Gd-enhancement of the lesion shown in C (white arrows)

**Fig. 2:**
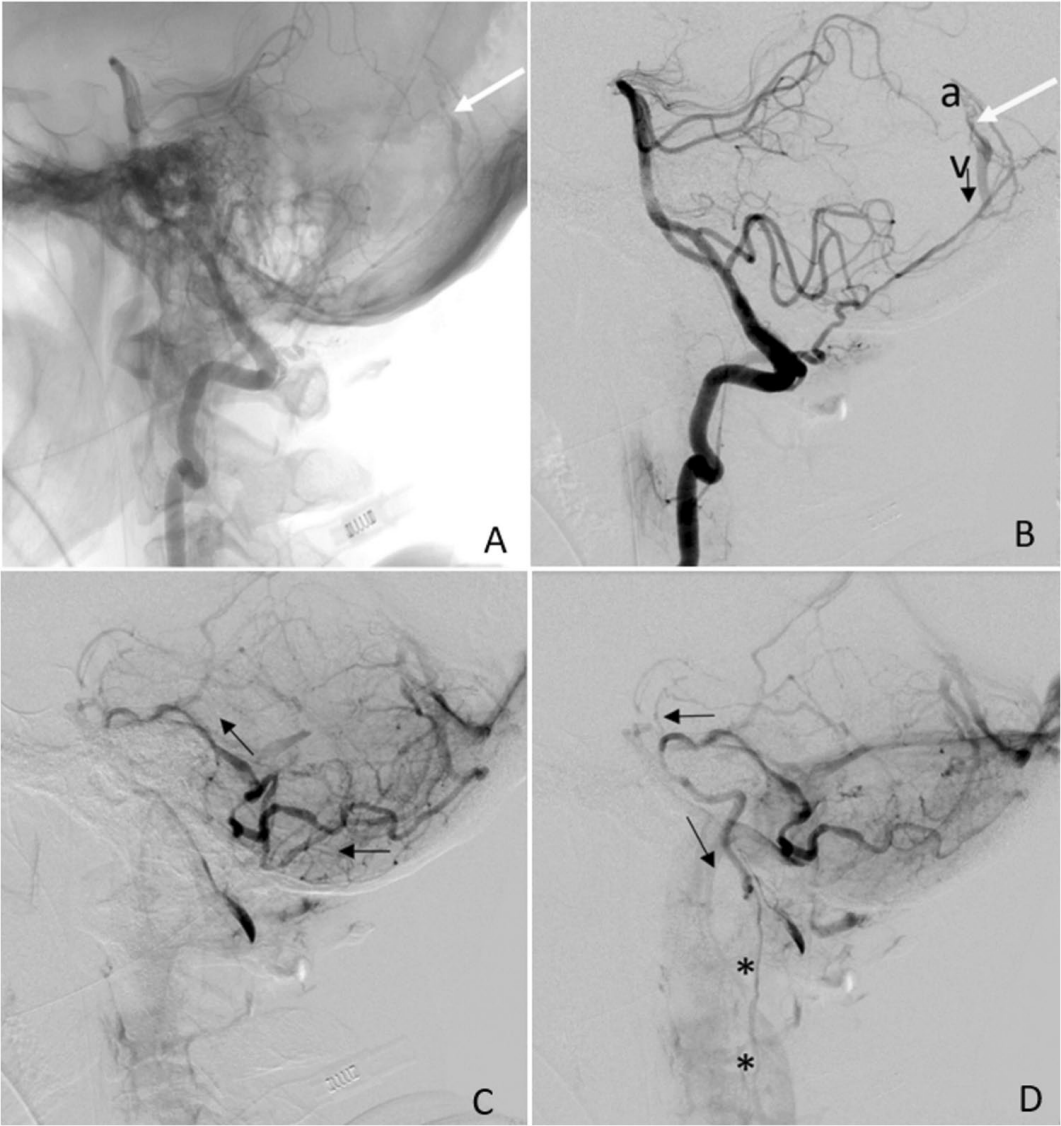
Cerebral angiography, vertebral artery injections.** A** The A-V shunt at the fistula site is indicated by the white arrow (LL view). The arterial feeder is the posterior meningeal artery (PMA), arising from the vertebral artery. **B** Same as in (A) but with digitally subtracted images showing the feeding artery (PMA, a), the fistula site (white arrow), and the precociously enhanced straight sinus (v). Retrograde venous drainage route is indicated by the black arrow. **C** Parenchimal**-**phase acquired image showing backward venous drainage route (black arrows) toward the perimedullary venous system.** D** Late acquisition image showing venous blood direction (black arrows) reaching the perimedullary venous system at cervical level (*)

In December 2019, a 52-year-old man presented with subacute onset of severe neck pain, vertigo, nausea, and vomiting which completely resolved within a month. In April 2020, he reported progressive difficulty in walking with tripping, climbing stairs, and manipulating small objects. He sought medical attention on May 1st when he experienced acute urinary retention requiring hospitalization and catheterization. He denied any history of fever, insect bites, trauma, or recent vaccinations. His past medical history included hypertension, gastroesophageal reflux disease, previous exposure to asbestos, and pulmonary fibrosis. There was no consanguinity between his parents, and family history was negative for neurological diseases. An urgent brain CT scan revealed a hypodense lesion in the right cerebellar hemisphere and subsequent MRI of the spine showed a gadolinium-enhancing spinal lesion suggestive of myelitis extending from the medulla to C7/D1. Cerebrospinal fluid (CSF) analysis was inconclusive, and search for common pathogens in the CSF was negative. The patient was started on high doses of steroids with mild clinical improvement. At discharge, his neurological examination showed left gaze evoked nystagmus, mild central left facial paresis, hyperreflexic quadriparesis with ankle clonus, abolished pain, and temperature and proprioception below D10 along with urinary and bowel incontinence. Despite initial improvement, the patient relapsed in August 2020 with worsening in his upper limb strength, increased lower limb spasticity, and altered mentation. MRI revealed extension of the previously described lesion to the pons (see Fig. 1). CFS analysis was once again uninformative and the extensive search even for tropical microorganisms was inconclusive. Vasculitides, neuromyelitis optica spectrum disorders, and other autoimmune systemic diseases were ruled out. A total-body PET study with 18-FDG did not show any findings consistent with neoplasm, and no malignant cells were found in the CSF. After a multidisciplinary discussion, neuroradiologists carefully reviewed the spinal MRI and identified the presence of tortuous vessels behind the cervical spinal cord. Subsequent cerebral angiography (digital subtraction angiography, DSA) confirmed the presence of an arterio-venous fistula between the posterior meningeal artery and the straight sinus with drainage into the perimedullary venous plexus at cervical level (Cognard type V fistula, see Fig. 2). The patient underwent endovascular treatment, which resulted in almost complete obliteration of the fistula; he was discharged to a rehabilitation center 2 weeks later. One year after the embolization, the patient’s neurological examination remained unchanged and he continued to use a wheelchair. In October 2021, he underwent successful retreatment with the endovascular approach, resulting in complete obliteration of the fistula, with only slight improvement in upper limb strength noted after the procedure.

## Materials and methods

### Literature search

We started identifying the published case reports and case series of patients having CVFs using a search strategy developed by three authors (ADG, ES, and CM) through an iterative process. We searched for published articles that mentioned iDAVF in title, abstract, and keywords using the following strategy: “Intracranial fistula AND spinal drainage,” “Intracranial fistula AND spinal cord,” “Intracranial fistula AND myelopathy,” and “Intracranial fistula AND myelitis,” since the inception of the database to March 2021. The language was restricted to English and Italian. The search was conducted independently by three experienced neurologists (ADG, ES, and CM) and was performed both (a) in PubMed electronic database and (b) through manual searches (i.e., reference lists of previously reported case reports/series and systematic reviews on this topic identified during the search).

### Study selection and data extraction

Following the procedure detailed by El Asri and colleagues and by Kamio et al. [10, 11], we collected all case reports published from inception to March 2021, thus providing a greater sample size of patients with CVF. We included all articles which (a) described a case or a series of cases of CVFs and that (b) were written in English or Italian. We excluded (1) articles in which full text could not be obtained; (2) papers concerning the pediatric populations; (3) unrelated papers (i.e., spinal fistulas).

The screening process was conducted as follows: first, the three authors (ADG, ES, and CM) independently reviewed all abstracts and titles for eligibility: after a manual screening, full-text reports were obtained if a study was deemed eligible or where eligibility was unclear. Then, reports were finally examined for inclusion, with disagreement resolved through consensus by the three authors.

Regarding the data extraction, we adopted a standardized coding scheme to collect data referring to (1) age and sex of patients, (2) type of CVF onset (acute, progressive, or multiphasic; see below), (3) presence of predisposing factors, (4) symptoms at onset (motor, sensory, sphincteric disturbances, ataxia, brainstem symptoms, dizziness/nausea/vomiting, and others), (5) symptoms at diagnosis, (6) time interval to definite diagnosis, (7) presence of an initial misdiagnosis, (8) MRI findings at diagnosis, (9) CVF localization, (10) feeding artery, (11) type of treatment (surgery or endovascular), (12) outcome (outlined as good recovery/complete regression, moderate disability, severe disability/death), (13) presence of a relapse, (14) length of post-treatment follow-up, and (15) angiography outcome. The authors coded all available information reported in any part of the articles, including tables and figures. Under “brainstem signs” we included the following: dysphagia, dysphonia, dysarthria, respiratory failure, diplopia, gaze-evoked nystagmus, decreased gag reflex, cranial nerves palsies, and hiccups. “Acute” onset was defined as an abrupt onset over 1 day or two, “multiphasic” onset was defined as bouts of symptoms with complete or almost complete recovery between the episodes, and “progressive” onset was preferred when disturbs developed over time with no definite *poussées*. For clinical outcome assessment, we defined “good recovery” (GR) as the complete regression of symptoms, “moderate disability” (MD) either as the ability to walk with assistance or neurologic sequalae interfering with daily activities but not determining loss of independence, and “severe disability (SD)/death” either as the inability to walk or as neurologic sequelae severely interfering with daily activities or as death. Missing data were not imputed.

### Statistical analysis

Data extracted from case series or case reports were then analyzed through descriptive statistics, including means, standard deviations, frequencies, and percentages.

Further, we tested several hypotheses on the association between socio-demographic, clinical, and neurological variables, and CVF (i) type of onset (acute, progressive, or multiphasic), (ii) outcomes (dichotomized as good outcome vs disability/death), (iii) time interval to diagnosis, and (iv) presence of specific symptoms at onset, through univariate and multivariate statistics. Differences between frequencies of specific categories were tested through chi square tests (with the significantly different categories identified through the adjusted standardized residuals >|2| [12]. Further, non-parametric correlations, Mann–Whitney U tests and Kruskal–Wallis ANOVAs were adopted to test for the association between dichotomous and continuous variables and for the presence of significant differences between groups on continuous independent variables, respectively.

All analyses were performed with SPSS 26 (IBM, 2019). All statistical tests were two tailed, and a *p* ≤ 0.05 was deemed statistically significant.

## Results

### Literature search and study inclusion

**Fig. 3:**
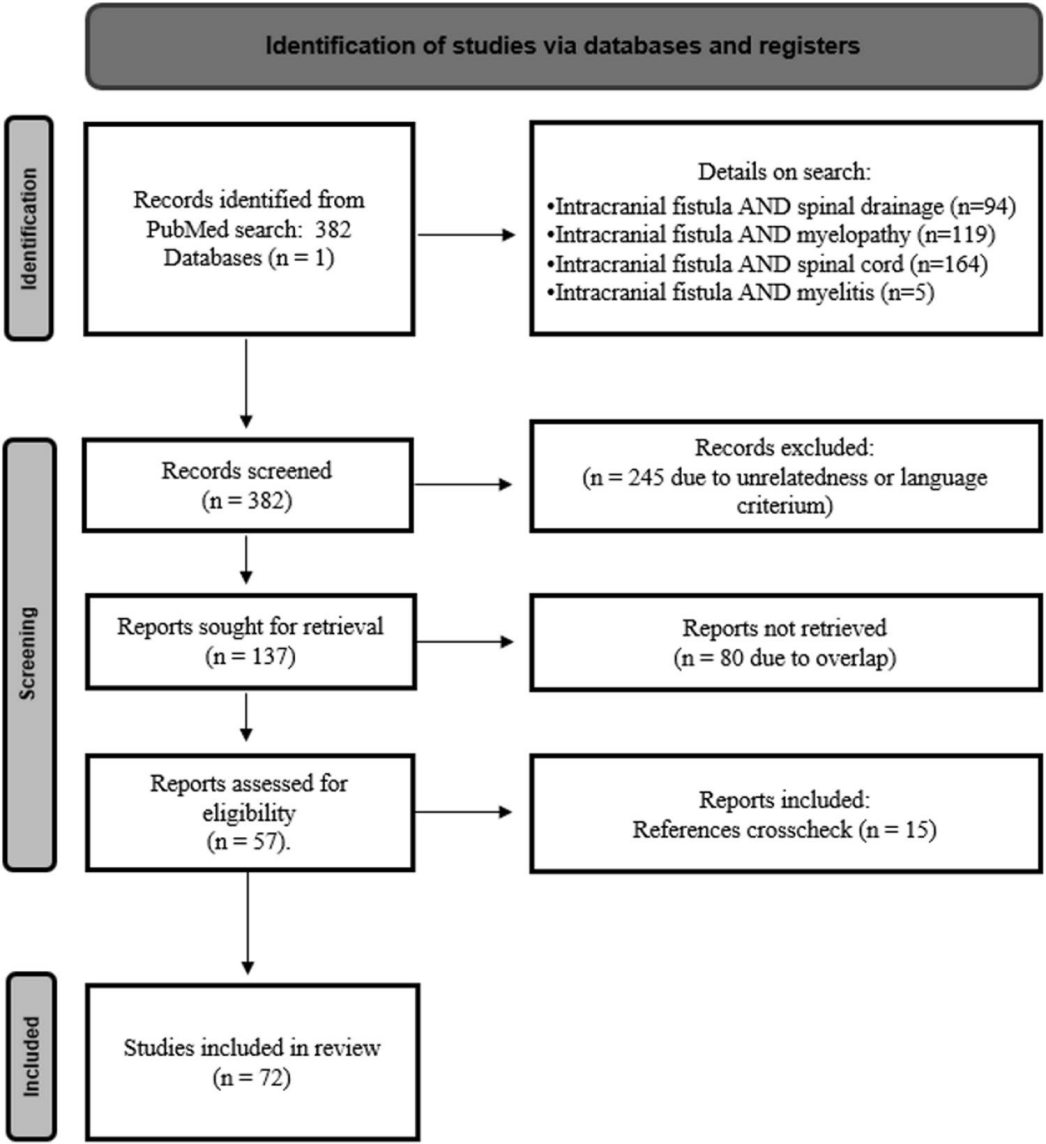
Flow chart of the searching strategy

The literature search initially yielded 382 articles. After screening titles and abstracts, 245 articles were excluded due to unrelatedness or because they were written in languages other than Italian or English. Additionally, 80 duplicates were removed, and 16 records were identified through other sources such as manual searches among reference lists of previously published reviews. The PRISMA flow diagram, depicted in Fig. 3, provides a graphical representation of the screening process. In total, 72 studies, including 60 case reports and 12 case series, were included in the analysis, providing data on a total of 100 patients with CVF. The median year of publication for the 72 studies was 2006, with a range from 1988 to 2020.

### Socio-demographic, clinical, and radiological features

**Fig. 4:**
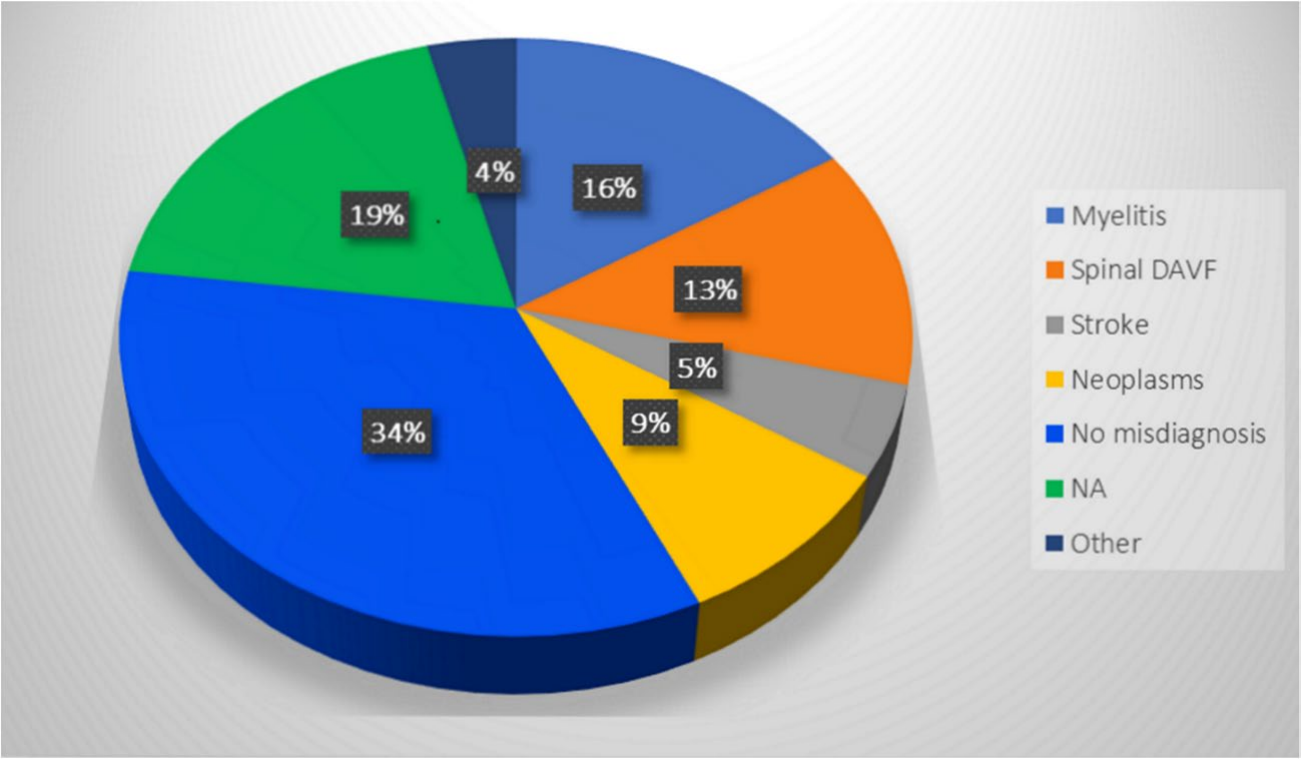
Misdiagnosis rate. *Note*. NA, not available

Table 2 summarizes the clinical characteristics of the patients with CVF. Most of the patients were middle aged, with a mean age of 56.20 ± 14.07 years and a range of 18–79 years and the majority were males (72%, *N* = 72). Predisposing factors, such as past head trauma, were reported in only 20.4% of the articles. The CVF’s onset was mostly progressive (*N* = 63, 64.9%), while multiphasic (*N* = 21, 21.6%) and acute (*N* = 13, 13.4%) onsets were less commonly reported. A total of 47 patients (58%) received an initial misdiagnosis. More details are provided in Fig. 4.

**Table 2:** Sociodemographic and clinical characteristics of patients with DAVF (*N* = 100)

Author	Year	Age	Sex	Onset	Predisposing factors	Interval to definite diagnosis	Initial misdiagnosis	Outcome
Abdelsadg et al.[2]	2016	65	F	Acute	No	0 months	No	GR
Abud et al.[23]	2015	66	F	Progressive	*NA*	1 month	No	GR
Aixut et al.[24]	2011	67	F	Multiphasic	*NA*	0 months	No	*NA*
Akkoc et al.[13]	2006	45	M	Progressive	*NA*	2 months	Stroke, transverse myelitis	SD
Asakawa et al.[25]	2002	64	M	Multiphasic	No	0.5 months	No	SD
Bernard et al.[17]	2018	65	F	Progressive	No	5 months	Neoplasm (glioma)	GR
Bousson et al.[26]	1999	36	M	Multiphasic	No	12 months	No	MD
Bret et al.[15]	1994	31	M	Multiphasic	No	4 months	Transverse myelitis	MD
Brunereau et al.[9] (1)	1996	35	F	Progressive	*NA*	*NA*	Spinal dural A-V fistula	*NA*
Brunereau et al.[9] (2)	1996	37	M	Progressive	*NA*	*NA*	Spinal dural A-V fistula	*NA*
Brunereau et al.[9] (3)	1996	53	M	Progressive	*NA*	*NA*	Spinal dural A-V fistula	*NA*
Brunereau et al.[9] (4)	1996	69	M	Progressive	*NA*	*NA*	Spinal dural A-V fistula	*NA*
Brunereau et al.[9] (5)	1996	68	F	Progressive	*NA*	*NA*	Spinal dural A-V fistula	*NA*
Brunereau et al.[9] (6)	1996	69	M	Progressive	*NA*	*NA*	Spinal dural A-V fistula	*NA*
Chen CJ et al.[27] (1)	1998	36	F	Progressive	*NA*	1 month	No	SD
Chen CJ et al.[27] (2)	1998	47	M	Progressive	Occipital skull fracture 2 years before	12 months	No	SD
Chen PM et al.[28]	2018	25	F	Acute	Occipital trauma 1 month prior	*NA*	Brainstem encephalitis, myelitis	*NA*
Chen PY et al.[29]	2019	66	M	Multiphasic	*NA*	1 month	Neoplasm	GR
Chng et al.[30]	2004	67	M	Acute	*NA*	0 months	No	MD
Clayton et al.[31]	2020	32	M	Progressive	No	1 month	Myelitis, GBS	MD
Copelan et al.[20] (1)	2018	59	M	Multiphasic	*NA*	1.25 months	Neoplasm	GR
Copelan et al.[20] (2)	2018	72	M	Progressive	Previous neurosurgery	3 months	NA	*NA*
Copelan et al.[20] (3)	2018	35	F	Progressive	Previous pilocytic astrocytoma	1 month	No	GR
Copelan et al.[20] (4)	2018	64	F	Progressive	*NA*	6 months	Transverse myelitis	SD
Deopujari et al.[32]	1995	50	F	Progressive	Intracranial hypertension/pseudotumor cerebri	6 months	No	GR
El Asri et al.[10]	2013	48	M	Acute	No history of trauma	0.3 months	Spinal dural A-V fistula	SD
Enokizono et al.[22] (1)	2017	60	M	Multiphasic	*NA*	7 months	*NA*	*NA*
Enokizono et al.[22](2)	2017	60	M	Progressive	*NA*	2 months	Transverse myelitis, Demyelinating lesion	*NA*
Ernst et al.[33] (1)	1997	71	M	Progressive	No	*NA*	No	MD
Ernst et al.[33] (2)	1997	47	M	Progressive	No	5 months	No	MD
Ernst et al.[33] (3)	1997	58	F	Progressive	No	*NA*	No	SD
Foreman et al.[34]	2013	59	M	Multiphasic	Muscular effort a few days before symptoms’ onset (moving boxes in his home); chiropractic manipulation the day of onset	0.75 months	Spinal cord trauma	SD
Gaensler et al.[35]	1989	50	M	Multiphasic	*NA*	48 months	*NA*	MD
Gobin et al.[36] (1)	1992	35	F	Progressive	*NA*	6 months	*NA*	GR
Gobin et al.[36] (2)	1992	37	M	Multiphasic	*NA*	9 months	*NA*	SD
Gobin et al.[36] (3)	1992	53	M	Progressive	Laminectomy	5 months	Cervical stenosis with spinal cord compression	SD
Gobin et al.[36] (4)	1992	69	M	Multiphasic	*NA*	12 months	*NA*	GR
Gobin et al.[36] (5)	1992	68	F	Progressive	*NA*	4 months	*NA*	MD
Gross et al.[37] (1)	2014	69	M	Acute	*NA*	5 days	GBS	GR
Gross et al.[37] (2)	2014	34	F	Progressive	Whole brain irradiation	0.25 months	Transverse myelitis	GR
Hähnel et al.[38]	1998	67	M	Progressive	No	6 months	No	GR
Haryu et al.[39]	2014	62	M	Progressive	No	4 months	Demyelinating lesion	MD
Iwase et al.[40]	2020	76	M	Acute	No	1 month	NMOSD	MD
Joseph et al.[41]	2000	42	M	Multiphasic	*NA*	24 months	Spinal cord infarction	MD
Jun Li et al.[18]	2004	73	M	Multiphasic	No	12 months	Stroke (twice)	MD
Kalamangalam et al.[21]	2002	68	M	Acute	No	4 months	Stroke	MD
Kamio et al.[11]	2015	66	F	Progressive	*NA*	8 months	Spinal dural A-V fistula	GR
Khan et al.[42]	2009	20	F	Acute	*NA*	0.5 months	Meningoencephalitis, NMOSD, sarcoidosis, Transverse myelitis	SD
Kim HJ et al.[43]	2015	61	M	Progressive	No	18 months	Myelitis, Neoplasm	SD
Kim NH et al.[44]	2011	45	M	Progressive	No	6 months	Demyelinating lesion	MD
Kim WY et al.[45]	2016	60	M	Progressive	*NA*	No delay (0 months)	Spinal dural A-V fistula	GR
Kleeberg et al.[46]	2010	60	M	Progressive	*NA*	*NA*	*NA*	*NA*
Kulwin et al.[47]	2012	44	F	Acute	No	*NA*	Stroke	SD
Kvint et al.[48]	2020	48	M	Multiphasic	No	6 months	Neoplasm	GR
Lagares et al.[49]	2007	65	M	Multiphasic	*NA*	3 months	Stroke	GR
Lv et al.[50]	2011	18	M	Progressive	*NA*	*NA*	*NA*	MD
Mascalchi et al. [51] (1)	1996	69	M	Progressive	Head trauma at age 25	48 months	No	*NA*
Mascalchi et al.[51] (2)	1996	53	M	Progressive	No	24 months	No	*NA*
Narita et al.[52]	1992	45	F	Acute	Previous treatment of CCF	0 months	No	GR
Ogbonnaya et al.[53]	2011	64	F	Progressive	No	3 months	No	NA
Pannu et al.[54]	2004	42	M	Progressive	No	12 months	No	MD
Partington et al.[55] (1)	1992	63	M	Progressive	*NA*	4 months	*NA*	GR
Partington et al.[55] (2)	1992	74	M	*NA*	*NA*	6 months	*NA*	SD
Patsalides et al.[16]	2010	53	M	Progressive	*NA*	*NA*	Neoplasm (lymphoma), encephalitis, demyelinating lesion	GR
Peethambar et al.[16]	2018	64	M	Progressive	No	1.5 months	Transverse myelitis	MD
Peltier et al.[56]	2011	58	F	Multiphasic	*NA*	2 months	*NA*	MD
Perkash et al.[57]	2002	79	M	Progressive	No	8 months	No	SD
Pop et al.[58]	2015	38	M	Multiphasic	No	2 months	Myelitis, GBS	MD
Renner et al.[59]	2006	58	M	Progressive	*NA*	*NA*	Spinal dural A-V fistula	GR
Ricolfi et al.[60] (1)	1998	69	M	Progressive	*NA*	36 months	No	SD
Ricolfi et al.[60] (2)	1998	53	M	Acute	*NA*	*NA*	No	MD
Ricolfi et al.[60] (3)	1998	40	F	Multiphasic	*NA*	0 months	No	SD
Ricolfi et al.[60] (4)	1998	75	F	Multiphasic	*NA*	*NA*	Transverse myelitis	GR
Ricolfi et al.[60] (5)	1998	51	F	*NA*	*NA*	5 months	Subarachnoid hemorrhage	GR
Rocca et al.[61]	2019	67	M	Progressive	*NA*	7 months	Transverse myelitis, neoplasm, spinal dural A-V fistula, TB, vasculitis, paraneoplastic syndrome, NMOSD, Lyme disease	SD
Rodriguez Rubio et al.[62]	2019	68	M	Acute	*NA*	*NA*	*NA*	MD
Roelz et al.[63]	2015	76	M	Multiphasic	No	8 months	Neoplasm, Demyelinating lesion	MD
Satoh et al.[64]	2005	38	F	Acute	No	0 months	Stroke	MD
Shimizu et al.[65]	2019	75	M	Progressive	No	6 months	No	MD
Singh et al.[66]	2013	*NA*	M	*NA*	No	5 months	Periodic paralysis, myelitis	GR
Sorenson et al.[67]	2019	57	M	Progressive	*NA*	*NA*	*NA*	*NA*
Sugiura et al.[68]	2009	69	F	Multiphasic	No	2 months	No	MD
Sun et al.[69]	2019	50	M	Progressive	*NA*	5 months	*NA*	GR
Tanaka et al.[70]	2017	64	M	Progressive	No	*NA*	No	MD
Tanoue et al.[71]	2005	70	M	Progressive	No	24 months	No	MD
Trop et al.[72]	1998	74	M	Progressive	No	12 months	No	MD
Tsutsumi et al.[73]	2008	62	F	Progressive	No	12 months	Neoplasm, myelitis	MD
Van Rooij et al.[74] (1)	2007	58	M	Progressive	*NA*	3 months	*NA*	GR
Van Rooij et al.[74] (2)	2007	65	M	Progressive	*NA*	12 months	*NA*	SD
Van Rooij et al.[74] (3)	2007	72	F	Progressive	*NA*	24 months	*NA*	SD
Versari et al.[75] (1)	1993	50	M	Progressive	No	7 months	No	MD
Versari et al.[75] (2)	1993	71	M	Progressive	No	9 months	No	GR
Wang et al.[76]	2019	57	M	Progressive	*NA*	3 months	No	GR
Wiesmann et al.[14]	2000	46	M	Multiphasic	*NA*	0.1 months	No	GR
Willinsky et al.[77]	1990	57	M	Progressive	No	36 months	No	SD
Wrobel et al.[78] (1)	1988	43	M	Progressive	*NA*	36 months	*NA*	MD
Wrobel et al.[78] (2)	1988	68	M	Progressive	*NA*	6 months	Spinal dural A-V fistula	SD
Wrobel et al.[78] (3)	1988	42	M	Progressive	*NA*	*NA*	Multiple sclerosis, Spinal dural A-V fistula, transverse myelitis	SD
Yoshida et al.[79]	1999	68	M	Progressive	No	6 months	No	MD
Zhang et al.[80]	2018	33	M	Progressive	No	2 months	Transverse myelitis	MD

*NA*, not available; *CCF*, carotid-cavernous fistula; *GBS*, Guillain-Barrè syndrome; *GR*, good recovery/complete remission; *MD*, moderate Disability; *NMOSD*, neuromyelitis optica spectrum disorders; *SD*, severe disability or death; *TB*, tuberculosis

**Fig. 5:**
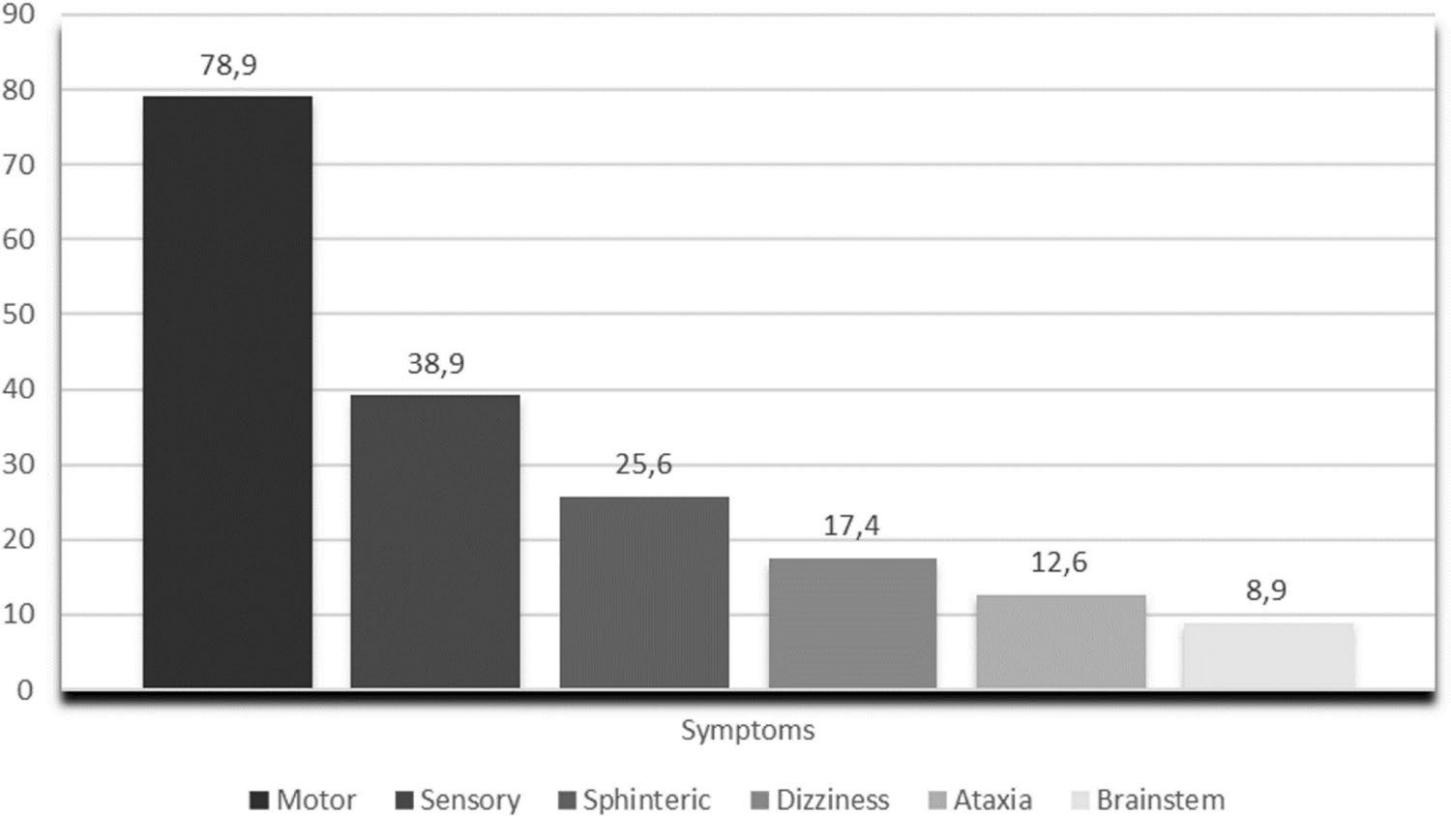
Symptoms at onset

The median time from symptoms onset to diagnosis was 5 months (range: 0–48 months). Figure 5 provides a graphical depiction of the prevalence of symptoms at onset.

MRI findings at diagnosis included flow voids (81.6%, *N* = 71), T2 hyperintensities (80.5%, *N* = 70), and swelling (56.3%, *N* = 49). DWI abnormalities, thrombosis, and T2* effects were rare (2 cases each, 2.3%), and contrast enhancement assessment was performed in only 55.8% of cases (*N* = 29).

As for clinical outcomes after treatment, 57 patients experienced a moderate-to-severe disability or died (*N* = 57; 67.1%; moderate disability, *N* = 33, 41.3%; severe disability/death = 19, 23.8%), while 28 experienced a partial-to-full recovery (32.9%).

Patients underwent endovascular treatment (*N* = 45, 48.9%), open surgery (*N* = 28, 30.4%), or both (*N* = 20, 21.3%); a total of 10 patients experienced a relapse after the first treatment attempt (11.9%).

Tables 2, 3, 4, 5, and 6 report all the clinical and radiological variables hitherto described.

**Table 3:** Additional clinical and neurological characteristics of patients with DAVF (*N* = 100)

Author	Year	DAVF localization	Feeding artery	Treatment	Relapse	Follow-up
Abdelsadg et al.[2]	2016	Left petrosal sinus	MHT, MMA	Endovascular	No	3 months
Abud et al.[23]	2015	Right sigmoid sinus	Right OA	Endovascular	No	3 months
Aixut et al.[24]	2011	Upper margin of the right petrosal bone	APhA	Endovascular	No	9 months
Akkoc et al.[13]	2006	Posterior fossa	Left OA, APhA	Endovascular (twice)	Yes	6 months
Asakawa et al.[25]	2002	CCJ	Left APhA	Endovascular + surgery	No	3 months
Bernard et al.[17]	2018	Right Jugular Foramen	Right APhA	Surgery	No	1 month
Bousson et al.[26]	1999	Tentorium cerebelli	Left OA	Endovascular	No	0,5 months
Bret et al.[15]	1994	Tentorium cerebelli	ICA (siphon)	Surgery	No	5 months
Brunereau et al.[9] (1)	1996	Left transverse sinus	Left MMA	*NA*	*NA*	*NA*
Brunereau et al.[9] (2)	1996	Left petrosal sinus	Left MMA	*NA*	*NA*	*NA*
Brunereau et al.[9] (3)	1996	Tentorium cerebelli	Left MHT	Surgery	*NA*	*NA*
Brunereau et al.[9] (4)	1996	Left petrosal sinus	Left APhA, left OA	*NA*	*NA*	*NA*
Brunereau et al.[9] (5)	1996	Left petrosal sinus	Left APA, left MMA, left OA	*NA*	*NA*	*NA*
Brunereau et al.[9] (6)	1996	Tentorium cerebelli	Left MHT	*NA*	*NA*	*NA*
Chen CJ et al.[27] (1)	1998	Torcular region	Left MMA, left VA	Surgery	No	3 months
Chen CJ et al.[27] (2)	1998	Torcular region	Left VA	Surgery	No	2 months
Chen PM et al.[28]	2018	Posterior fossa	Right VA	Endovascular	No	After the embolization
Chen PY et al.[29]	2019	*NA*	Right OA, right distal VA	Endovascular	No	3 months
Chng et al.[30]	2004	CCJ	Right APhA	Endovascular	No	2 days
Clayton et al.[31]	2020	Petrous apex	Cavernous ICA	Endovascular + surgery	Yes	48 months
Copelan et al.[20] (1)	2018	Left superior petrosal sinus	OA, APhA, MMA	Endovascular + surgery	No	36 months
Copelan et al.[20] (2)	2018	Anterior condilar vein	Right APhA	Endovascular	No	5 months
Copelan et al.[20] (3)	2018	Superior petrosal sinus	OA	Endovascular	No	3 months
Copelan et al.[20] (4)	2018	Superior petrosal sinus	IFLT	Endovascular + surgery	Yes	12 months
Deopujari et al.[32]	1995	Overlying the right cerebellar hemisphere	MMA, OA	Endovascular + surgery	No	1 month
El Asri et al.[10]	2013	Left tentorial (posterior fossa)	Tentorial artery of Bernasconi and Cassinari	Surgery	No	2 months
Enokizono et al.[22] (1)	2017	Tentorium cerebelli	Right MHT, MMA and AMA	Surgery	No	*NA*
Enokizono et al.[22](2)	2017	Tentorium cerebelli	MMA	Endovascular + surgery	No	*NA*
Ernst et al.[33] (1)	1997	Superior Petrosal sinus	*NA*	Surgery	No	18 months
Ernst et al.[33] (2)	1997	Occipital condyle	Right APhA	Endovascular	No	48 months
Ernst et al.[33] (3)	1997	Skull Base	Ascending cervical, vertebral, ophthalmic	Endovascular	Yes	48 months
Foreman et al.[34]	2013	CCJ	MHT	Surgery	No	*NA*
Gaensler et al.[35]	1989	Anterior foramen magnum	VA and APhA	Endovascular	No	24 months
Gobin et al.[36] (1)	1992	Left lateral sinus	MMA and OA	Endovascular	No	6 months
Gobin et al.[36] (2)	1992	Left petrous apex	MMA	Endovascular + surgery	NV	No follow-up (death)
Gobin et al.[36] (3)	1992	Left tentorium cerebelli	MHT	Endovascular	No	6 months
Gobin et al.[36] (4)	1992	Left superior petrous sinus	APhA and OA	Endovascular + surgery	No	12 months
Gobin et al.[36] (5)	1992	Left superior petrous sinus	Left MMA, APhA, OA	Endovascular	No	8 months
Gross et al.[37] (1)	2014	Posterior fossa	Left MMA, left ICA, OA and PA	Endovascular	No	2,5 months
Gross et al.[37] (2)	2014	Left transverse sigmoid junction	Left OA	Endovascular	No	3 months
Hähnel et al.[38]	1998	*NA*	APhA, OA	Endovascular	No	2.5 months
Haryu et al.[39]	2014	Tentorium cerebelli	MMA	Surgery	No	*NA*
Iwase et al.[40]	2020	*NA*	OA	Endovascular + surgery	No	2 months
Joseph et al.[41]	2000	*NA*	Left MMA, PMA, and both OA	Endovascular	No	2 months
Jun Li et al.[18]	2004	Left transverse sinus	Left MMA, OA, right APhA	Endovascular	No	5 days
Kalamangalam et al.[21]	2002	Clivus	ICA	Surgery	No	4 months
Kamio et al.[11]	2015	Left transverse-sigmoid sinus	Left OA, MMA	Endovascular	No	3 months
Khan et al.[42]	2009	Left tentorium cerebelli	Tentorial artery of Bernasconi and Cassinari	Surgery	No	3 months
Kim HJ et al.[43]	2015	Petrous ridge	MMA	Endovascular	No	6 months
Kim NH et al.[44]	2011	Left petrous region	ICA	Surgery	No	1 month
Kim WY et al.[45]	2016	Posterior fossa (prepontine vein)	MHT, artery of foramen rotundum, right MMA	Endovascular	No	12 months
Kleeberg et al.[46]	2010	Tentorium cerebelli	Tentorial artery of Bernasconi and Cassinari	Endovascular + surgery	*NA*	*NA*
Kulwin et al.[47]	2012	Superior Petrosal sinus	MMA, VA	Surgery	No	*NA*
Kvint et al.[48]	2020	Tentorium cerebelli	SCA	Surgery	No	3 months
Lagares et al.[49]	2007	Torcular Herophilii	*NA*	Surgery	No	6 months
Lv et al.[50]	2011	Tentorium cerebelli	Left MHT, MMA	Endovascular	No	5 months
Mascalchi et al. [51] (1)	1996	Skull base	APhA, VA	Surgery	*NA*	*NA*
Mascalchi et al.[51] (2)	1996	Condylar channel	APhA	Endovascular	*NA*	*NA*
Narita et al.[52]	1992	CCF	VA, ICA	Surgery	No*	2 months
Ogbonnaya et al.[53]	2011	*NA*	*NA*	Endovascular	*NA*	*NA*
Pannu et al.[54]	2004	Right tentorium cerebelli	Cavernous segment of ICA	Endovascular + surgery	No	12 months
Partington et al.[55] (1)	1992	Left foramen magnum	PMA	Surgery	No	9 months
Partington et al.[55] (2)	1992	Right foramen magnum	PMA	None (died)	*NA*	*NA*
Patsalides et al.[16]	2010	Superior petrosal sinus	MHT, MMA	Endovascular	No	9 months
Peethambar et al.[16]	2018	Left tentorium cerebelli	Tentorial artery of Bernasconi and Cassinari	Endovascular + surgery	No	3 months
Peltier et al.[56]	2011	CCJ	Left VA	Endovascular + surgery	No	6 months
Perkash et al.[57]	2002	Petrous apex	VA, APhA, PA	None (refused)	*NA*	*NA*
Pop et al.[58]	2015	Foramen magnum	OA, APhA	Endovascular	Yes	6 months
Renner et al.[59]	2006	Tentorium cerebelli	Right MHT	Surgery	No	3 months
Ricolfi et al.[60] (1)	1998	Tentorium cerebelli	Artery of foramen rotundum, C5 ICA	Endovascular	*NA*	*NA*
Ricolfi et al.[60] (2)	1998	Tentorium cerebelli	MMA and C5 ICA	Endovascular + surgery	Yes	24 months
Ricolfi et al.[60] (3)	1998	Right cavernous sinus	ICA and ECA	Endovascular twice	Yes	*NA*
Ricolfi et al.[60] (4)	1998	Left superior petrosal sinus	Left MMA, OA, right APhA	Endovascular	No	60 months
Ricolfi et al.[60] (5)	1998	Right sigmoid sinus	Right OA, MMA	Endovascular	No	12 months
Rocca et al.[61]	2019	Right lateral region of foramen magnum	APhA	Surgery	No	*NA*
Rodriguez Rubio et al.[62]	2019	Tentorium cerebelli	Right PMA	Endovascular + surgery	*NA*	No follow-up
Roelz et al.[63]	2015	Petrous ridge	MMA, APhA, OA	Endovascular + surgery	Yes	6 months after first treatment and 0.5 months after the 2nd one
Satoh et al.[64]	2005	Left tranverse-sigmoid sinus	Right MMA, OA, APhA, VA, Left MHT	Endovascular	No	1 month
Shimizu et al.[65]	2019	Anterior cranial fossa	Anterior ethmoidal artery	Surgery	No	2 months
Singh et al.[66]	2013	Left tentorium cerebelli	MMA, ICA	Endovascular + surgery	No	*NA*
Sorenson et al.[67]	2019	CCJ	PICA	Endovascular + surgery	Yes	*NA*
Sugiura et al.[68]	2009	Sigmoid sinus and superior petrosal sinus	OA	Endovascular	No	0.75 months
Sun et al.[69]	2019	Foramen magnum	Left VA	Surgery	No	0.3 months
Tanaka et al.[70]	2017	Occipital sinus	PMA	Endovascular	No	8 months
Tanoue et al.[71]	2005	Anterior condylar vein	APhA, OA	Endovascular	No	12 months
Trop et al.[72]	1998	Foramen magnum	VA	Surgery	No	*NA*
Tsutsumi et al.[73]	2008	Petrosal and cavernous sinus	APhA and OA	Endovascular	No	*NA*
Van Rooij et al.[74] (1)	2007	Tentorium cerebelli	APhA, MMA	Endovascular	No	12 months
Van Rooij et al.[74] (2)	2007	Petrous ridge	Stylomastoid artery	Endovascular + surgery	No	12 months
Van Rooij et al.[74] (3)	2007	Marginal sinus of the foramen magnum	OA	Endovascular	No	24 months
Versari et al.[75] (1)	1993	Superior Petrosal sinus	MHT	Surgery	No	24 months
Versari et al.[75] (2)	1993	Sigmoid sinus	OA, MMA	Endovascular + surgery	No	6 months
Wang et al.[76]	2019	Dorsal sellae	Right MHT, ophthalmic artery, MMA	Endovascular	Yes	24 months
Wiesmann et al.[14]	2000	Anteromedian pontine vein	Left APhA	Endovascular	No	12 months
Willinsky et al.[77]	1990	Foramen magnum	APhA	Endovascular	No	18 months
Wrobel et al.[78] (1)	1988	Right petrous apex	OA, ICA	Endovascular	No	9 months
Wrobel et al.[78] (2)	1988	Petrous apex	OA, ICA	Surgery	No	3 months
Wrobel et al.[78] (3)	1988	Tentorium cerebelli	MHT, OA, APhA	Surgery	No	3 months
Yoshida et al.[79]	1999	CCJ	VA	Surgery	No	*NA*
Zhang et al.[80]	2018	*NA*	MHT	Endovascular	No	1 month

*NA*, not available; *APhA*, ascending pharyngeal artery; *CCJ*, cranio-cervical junction; *CCF*, carotid-cavernous fistula; *ECA*, external carotid artery; *ICA*, internal carotid artery; *IFLT*, inferolateral trunk; *MHT*, meningohypophyseal trunk; *MMA*, middle meningeal artery; *OA*, occipital artery; *PA*, posterior auricular; *PICA*, posterior inferior cerebellar artery; *PMA*, posterior meningeal artery; *SCA*, superior cerebellar artery; *VA*, vertebral artery

^*^This episode itself is a relapse

**Table 4:** Symptoms at onset among patients with DAVF (*N* = 100)

Author	Year	Motor	Sensory	Sphincteric disturbance	Ataxia	Brainstem symptoms	Dizziness, nausea, vomiting	Other
Abdelsadg et al.[2]	2016	Yes	No	Yes	No	No	Yes	Vertigo
Abud et al.[23]	2015	Yes	No	No	No	No	No	No
Aixut et al.[24]	2011	Yes	No	Yes	No	No	No	Acute neck pain
Akkoc et al.[13]	2006	Yes	No	Yes	No	No	Yes	Occipital headache
Asakawa et al.[25]	2002	Yes	No	No	No	No	No	No
Bernard et al.[17]	2018	*NA*	*NA*	*NA*	*NA*	*NA*	*NA*	*NA*
Bousson et al.[26]	1999	No	Yes	No	No	No	No	No
Bret et al.[15]	1994	Yes	No	No	No	No	No	No
Brunereau et al.[9] (1)	1996	Yes	Yes	No	No	No	No	No
Brunereau et al.[9] (2)	1996	Yes	Yes	No	No	No	No	No
Brunereau et al.[9] (3)	1996	Yes	Yes	No	No	No	No	No
Brunereau et al.[9] (4)	1996	Yes	Yes	No	No	No	No	No
Brunereau et al.[9] (5)	1996	Yes	Yes	No	No	No	No	No
Brunereau et al.[9] (6)	1996	Yes	Yes	No	No	No	No	No
Chen CJ et al.[27] (1)	1998	Yes	Yes	No	No	No	No	No
Chen CJ et al.[27] (2)	1998	Yes	No	Yes	No	No	No	No
Chen PM et al.[28]	2018	Yes	Yes	No	No	Yes	No	No
Chen PY et al.[29]	2019	Yes	No	No	No	No	Yes	Vertigo
Chng et al.[30]	2004	Yes	No	No	No	No	No	No
Clayton et al.[31]	2020	Yes	No	Yes	*NA*	No	No	No
Copelan et al.[20] (1)	2018	No	No	No	No	No	Yes	Vertigo
Copelan et al.[20] (2)	2018	No	No	No	Yes	Yes	No	No
Copelan et al.[20] (3)	2018	Yes	No	No	No	Yes	No	No
Copelan et al.[20] (4)	2018	Yes	No	No	No	No	No	No
Deopujari et al.[32]	1995	Yes	Yes	No	Yes	*NA*	No	No
El Asri et al.[10]	2013	Yes	No	No	No	No	No	No
Enokizono et al.[22] (1)	2017	Yes	Yes	Yes	No	Yes	No	No
Enokizono et al.[22](2)	2017	Yes	Yes	No	*NA*	Yes	No	No
Ernst et al.[33] (1)	1997	No	No	No	No	No	Yes	No
Ernst et al.[33] (2)	1997	*NA*	*NA*	*NA*	*NA*	*NA*	*NA*	*NA*
Ernst et al.[33] (3)	1997	*NA*	*NA*	*NA*	*NA*	*NA*	*NA*	*NA*
Foreman et al.[34]	2013	No	Yes	No	No	No	No	Cervical and lumbar pain
Gaensler et al.[35]	1989	Yes	No	No	No	No	No	No
Gobin et al.[36] (1)	1992	Yes	No	No	No	No	No	Headache, ear bruit
Gobin et al.[36] (2)	1992	Yes	No	No	No	No	No	No
Gobin et al.[36] (3)	1992	Yes	Yes	No	No	No	No	Lumbar and upper extremities pain
Gobin et al.[36] (4)	1992	Yes	No	No	No	No	No	No
Gobin et al.[36] (5)	1992	Yes	No	No	No	No	No	Headache
Gross et al.[37] (1)	2014	Yes	Yes	Yes	No	No	No	No
Gross et al.[37] (2)	2014	Yes	No	No	No	No	No	No
Hähnel et al.[38]	1998	*NA*	*NA*	*NA*	*NA*	*NA*	*NA*	*NA*
Haryu et al.[39]	2014	Yes	No	Yes	*NA*	No	No	No
Iwase et al.[40]	2020	Yes	No	No	No	No	No	No
Joseph et al.[41]	2000	Yes	No	No	No	No	No	Lumbar pain
Jun Li et al.[18]	2004	Yes	No	Yes	No	Yes	Yes	No
Kalamangalam et al.[21]	2002	Yes	No	No	Yes	No	No	Dizziness
Kamio et al.[11]	2015	No	Yes	No	No	No	No	No
Khan et al.[42]	2009	Yes	Yes	Yes	No	No	Yes	No
Kim HJ et al.[43]	2015	Yes	Yes	No	No	No	No	No
Kim NH et al.[44]	2011	Yes	Yes	No	No	No	No	No
Kim WY et al.[45]	2016	Yes	No	No	No	No	No	No
Kleeberg et al.[46]	2010	Yes	No	No	No	No	No	No
Kulwin et al.[47]	2012	Yes	No	Yes	No	No	No	Altered mental status
Kvint et al.[48]	2020	Yes	No	No	No	No	No	No
Lagares et al.[49]	2007	No	No	No	No	No	Yes	No
Lv et al.[50]	2011	Yes	Yes	Yes	No	No	No	No
Mascalchi et al. [51] (1)	1996	*NA*	*NA*	*NA*	*NA*	*NA*	*NA*	*NA*
Mascalchi et al.[51] (2)	1996	No	Yes	No	No	No	No	No
Narita et al.[52]	1992	Yes	No	No	No	No	No	No
Ogbonnaya et al.[53]	2011	Yes	No	No	No	No	No	No
Pannu et al.[54]	2004	No	No	No	Yes	No	Yes	No
Partington et al.[55] (1)	1992	Yes	Yes	Yes	No	No	No	Erectile dysfunction
Partington et al.[55] (2)	1992	*NA*	*NA*	*NA*	*NA*	*NA*	No	*NA*
Patsalides et al.[16]	2010	No	Yes	No	No	No	No	Drop attacks
Peethambar et al.[16]	2018	Yes	Yes	Yes	No	No	No	Erectile dysfunction
Peltier et al.[56]	2011	Yes	No	No	No	No	No	Occipital neuralgia
Perkash et al.[57]	2002	*NA*	*NA*	*NA*	*NA*	No	No	*NA*
Pop et al.[58]	2015	No	No	No	No	No	No	Seizure (GTCS)
Renner et al.[59]	2006	Yes	Yes	Yes	Yes	No	No	No
Ricolfi et al.[60] (1)	1998	Yes	No	Yes	No	No	No	Erectile dysfunction, left ear bruit, postural hypotension
Ricolfi et al.[60] (2)	1998	Yes	Yes	Yes	No	No	No	No
Ricolfi et al.[60] (3)	1998	Yes	No	No	No	No	Yes	Right exophthalmos, conjunctival hyperaemia, headache
Ricolfi et al.[60] (4)	1998	Yes	No	Yes	No	Yes	No	Dysautonomia
Ricolfi et al.[60] (5)	1998	No	No	No	No	No	Yes	Headache
Rocca et al.[61]	2019	Yes	Yes	No	Yes	No	No	No
Rodriguez Rubio et al.[62]	2019	Yes	No	No	No	No	No	No
Roelz et al.[63]	2015	No	No	No	Yes	Yes	Yes	No
Satoh et al.[64]	2005	No	No	No	No	No	Yes	No
Shimizu et al.[65]	2019	Yes	No	No	Yes	No	Yes	No
Singh et al.[66]	2013	Yes	No	No	No	No	Yes	No
Sorenson et al.[67]	2019	*NA*	*NA*	*NA*	*NA*	*NA*	*NA*	*NA*
Sugiura et al.[68]	2009	No	No	No	No	No	No	Pulsatile tinnitus
Sun et al.[69]	2019	*NA*	*NA*	*NA*	*NA*	*NA*	*NA*	*NA*
Tanaka et al.[70]	2017	Yes	No	No	No	No	No	No
Tanoue et al.[71]	2005	No	Yes	No	No	No	No	No
Trop et al.[72]	1998	*NA*	*NA*	*NA*	*NA*	*NA*	*NA*	*NA*
Tsutsumi et al.[73]	2008	No	No	No	No	No	No	Tinnitus, occipital neuralgia
Van Rooij et al.[74] (1)	2007	Yes	No	Yes	No	No	No	No
Van Rooij et al.[74] (2)	2007	Yes	No	Yes	No	No	No	No
Van Rooij et al.[74] (3)	2007	Yes	Yes	Yes	No	No	No	No
Versari et al.[75] (1)	1993	Yes	No	No	No	No	No	No
Versari et al.[75] (2)	1993	Yes	No	No	Yes	No	No	Brachialgia
Wang et al.[76]	2019	No	Yes	No	No	No	No	No
Wiesmann et al.[14]	2000	Yes	No	Yes	Yes	No	Yes	Occipital neuralgia
Willinsky et al.[77]	1990	No	Yes	No	No	No	No	Chest pain
Wrobel et al.[78] (1)	1988	Yes	Yes	No	Yes	No	No	No
Wrobel et al.[78] (2)	1988	Yes	Yes	No	No	No	No	Spasm
Wrobel et al.[78] (3)	1988	Yes	Yes	Yes	No	No	No	No
Yoshida et al.[79]	1999	Yes	Yes	No	No	No	No	No
Zhang et al.[80]	2018	Yes	No	No	No	No	No	No

*NA*, not available; *GTCS*, generalized tonic–clonic seizure

**Table 5:** Symptoms at diagnosis among patients with DAVF (*N* = 100)

Author	Year	Motor	Sensory	Sphincteric disturbance	Ataxia	Brainstem symptoms	Dizziness, nausea, vomiting	Other
Abdelsadg et al.[2]	2016	Yes	No	Yes	Yes	No	No	Vertigo
Abud et al.[23]	2015	Yes	No	No	No	No	No	No
Aixut et al.[24]	2011	Yes	No	Yes	No	No	No	No
Akkoc et al.[13]	2006	Yes	No	Yes	No	No	No	No
Asakawa et al.[25]	2002	Yes	No	Yes	No	No	No	No
Bernard et al.[17]	2018	No	No	No	Yes	Yes	No	Tinnitus
Bousson et al.[26]	1999	Yes	Yes	No	No	No	No	No
Bret et al.[15]	1994	Yes	Yes	Yes	No	No	No	No
Brunereau et al.[9] (1)	1996	Yes	Yes	No	No	Yes	No	No
Brunereau et al.[9] (2)	1996	Yes	Yes	No	No	Yes	No	No
Brunereau et al.[9] (3)	1996	Yes	Yes	No	No	Yes	No	No
Brunereau et al.[9] (4)	1996	Yes	Yes	No	No	No	No	No
Brunereau et al.[9] (5)	1996	Yes	Yes	No	No	No	No	No
Brunereau et al.[9] (6)	1996	Yes	Yes	No	No	No	No	No
Chen CJ et al.[27] (1)	1998	Yes	Yes	Yes	No	No	No	No
Chen CJ et al.[27] (2)	1998	Yes	No	Yes	No	No	No	Erectile dysfunction
Chen PM et al.[28]	2018	Yes	Yes	No	No	Yes	No	No
Chen PY et al.[29]	2019	Yes	No	No	No	No	Yes	Vertigo
Chng et al.[30]	2004	Yes	No	No	No	No	No	No
Clayton et al.[31]	2020	Yes	No	NV	No	Yes	No	No
Copelan et al.[20] (1)	2018	*NA*	*NA*	*NA*	*NA*	*NA*	*NA*	*NA*
Copelan et al.[20] (2)	2018	No	No	No	Yes	Yes	No	No
Copelan et al.[20] (3)	2018	*NA*	*NA*	*NA*	*NA*	*NA*	*NA*	*NA*
Copelan et al.[20] (4)	2018	Yes	No	No	No	No	No	No
Deopujari et al.[32]	1995	Yes	Yes	Yes	Yes	Yes	No	No
El Asri et al.[10]	2013	Yes	Yes	No	No	Yes	No	No
Enokizono et al.[22] (1)	2017	*NA*	*NA*	*NA*	*NA*	*NA*	*NA*	*NA*
Enokizono et al.[22](2)	2017	Yes	Yes	No	*NA*	Yes	No	No
Ernst et al.[33] (1)	1997	Yes	No	No	No	No	No	No
Ernst et al.[33] (2)	1997	*NA*	*NA*	*NA*	*NA*	*NA*	*NA*	*NA*
Ernst et al.[33] (3)	1997	*NA*	*NA*	*NA*	*NA*	*NA*	*NA*	*NA*
Foreman et al.[34]	2013	Yes	Yes	Yes	No	No	No	No
Gaensler et al.[35]	1989	Yes	Yes	Yes	No	No	No	Erectile dysfunction
Gobin et al.[36] (1)	1992	Yes	No	No	No	Yes	No	No
Gobin et al.[36] (2)	1992	Yes	No	No	No	Yes	No	No
Gobin et al.[36] (3)	1992	Yes	Yes	No	No	No	No	No
Gobin et al.[36] (4)	1992	Yes	No	No	No	No	No	No
Gobin et al.[36] (5)	1992	Yes	Yes	No	No	Yes	No	Cervical pain
Gross et al.[37] (1)	2014	Yes	Yes	Yes	No	No	No	No
Gross et al.[37] (2)	2014	Yes	No	No	No	No	No	No
Hähnel et al.[38]	1998	Yes	No	No	No	No	No	No
Haryu et al.[39]	2014	Yes	No	Yes	Yes	Yes	No	No
Iwase et al.[40]	2020	Yes	No	No	No	Yes	No	No
Joseph et al.[41]	2000	Yes	Yes	Yes	No	No	No	No
Jun Li et al.[18]	2004	Yes	No	Yes	No	No	No	No
Kalamangalam et al.[21]	2002	Yes	No	Yes	Yes	Yes	No	No
Kamio et al.[11]	2015	No	Yes	No	No	No	No	No
Khan et al.[42]	2009	Yes	Yes	Yes	No	Yes	No	No
Kim HJ et al.[43]	2015	Yes	Yes	Yes	No	No	No	No
Kim NH et al.[44]	2011	Yes	Yes	No	No	Yes	No	No
Kim WY et al.[45]	2016	Yes	No	No	No	No	No	No
Kleeberg et al.[46]	2010	Yes	No	No	No	No	No	No
Kulwin et al.[47]	2012	Yes	No	Yes	No	Yes	No	No
Kvint et al.[48]	2020	Yes	No	No	No	No	No	No
Lagares et al.[49]	2007	Yes	No	No	No	Yes	No	No
Lv et al.[50]	2011	Yes	Yes	Yes	No	No	No	No
Mascalchi et al. [51] (1)	1996	Yes	Yes	Yes	No	No	No	No
Mascalchi et al.[51] (2)	1996	Yes	Yes	No	No	No	No	No
Narita et al.[52]	1992	Yes	No	No	No	No	No	No
Ogbonnaya et al.[53]	2011	Yes	No	No	Yes	No	No	No
Pannu et al.[54]	2004	Yes	No	Yes	Yes	No	No	No
Partington et al.[55] (1)	1992	Yes	Yes	Yes	No	No	No	No
Partington et al.[55] (2)	1992	Yes	Yes	Yes	No	No	No	No
Patsalides et al.[16]	2010	No	Yes	No	Yes	No	No	No
Peethambar et al.[16]	2018	Yes	Yes	Yes	No	No	No	No
Peltier et al.[56]	2011	Yes	Yes	Yes	No	Yes	No	No
Perkash et al.[57]	2002	Yes	Yes	Yes	No	No	No	No
Pop et al.[58]	2015	Yes	Yes	Yes	No	No	No	No
Renner et al.[59]	2006	Yes	Yes	Yes	Yes	No	No	No
Ricolfi et al.[60] (1)	1998	Yes	Yes	Yes	No	No	No	No
Ricolfi et al.[60] (2)	1998	Yes	Yes	Yes	No	No	No	No
Ricolfi et al.[60] (3)	1998	Yes	No	Yes	Yes	Yes	No	No
Ricolfi et al.[60] (4)	1998	Yes	Yes	Yes	No	Yes	No	No
Ricolfi et al.[60] (5)	1998	Yes	No	Yes	No	Yes	No	Postural hypotension
Rocca et al.[61]	2019	Yes	Yes	Yes	Yes	No	No	No
Rodriguez Rubio et al.[62]	2019	Yes	No	No	No	No	No	No
Roelz et al.[63]	2015	No	No	No	Yes	Yes	Yes	Blurred vision
Satoh et al.[64]	2005	Yes	Yes	No	No	Yes	No	No
Shimizu et al.[65]	2019	Yes	Yes	No	Yes	No	No	No
Singh et al.[66]	2013	Yes	No	No	Yes	No	No	No
Sorenson et al.[67]	2019	*NA*	*NA*	*NA*	*NA*	*NA*	*NA*	*NA*
Sugiura et al.[68]	2009	No	No	No	Yes	Yes	Yes	No
Sun et al.[69]	2019	Yes	Yes	Yes	No	No	No	No
Tanaka et al.[70]	2017	Yes	No	Yes	No	No	No	No
Tanoue et al.[71]	2005	Yes	Yes	No	NV	No	No	No
Trop et al.[72]	1998	Yes	No	No	No	No	No	No
Tsutsumi et al.[73]	2008	Yes	Yes	Yes	No	No	No	No
Van Rooij et al.[74] (1)	2007	Yes	Yes	Yes	No	No	No	No
Van Rooij et al.[74] (2)	2007	Yes	No	Yes	No	No	No	No
Van Rooij et al.[74] (3)	2007	Yes	Yes	Yes	No	No	No	No
Versari et al.[75] (1)	1993	Yes	Yes	No	No	Yes	No	No
Versari et al.[75] (2)	1993	Yes	No	Yes	Yes	No	No	No
Wang et al.[76]	2019	No	Yes	No	No	No	No	No
Wiesmann et al.[14]	2000	Yes	No	Yes	Yes	Yes	No	No
Willinsky et al.[77]	1990	Yes	Yes	Yes	No	No	No	No
Wrobel et al.[78] (1)	1988	Yes	Yes	No	Yes	No	No	No
Wrobel et al.[78] (2)	1988	Yes	Yes	No	No	No	No	No
Wrobel et al.[78] (3)	1988	Yes	Yes	Yes	No	No	No	No
Yoshida et al.[79]	1999	Yes	Yes	Yes	No	Yes	No	No
Zhang et al.[80]	2018	Yes	No	No	No	No	No	No

*NA*, not available

**Table 6:** Brain MRI findings at diagnosis among patients with DAVF (*N* = 100)

Author	Year	Swelling	Hyper T2	Flow voids or abnormal vessels	Contrast enhancement	DWI abnormality	Thrombosis	T2* effects
Abdelsadg et al.[2]	2016	Yes	Yes	No	No	Yes	No	No
Abud et al.[23]	2015	Yes	Yes	Yes	No	No	No	No
Aixut et al.[24]	2011	Yes	Yes	Yes	No	No	Yes	No
Akkoc et al.[13]	2006	*NA*	Yes	Yes	No	No	No	No
Asakawa et al.[25]	2002	Yes	Yes	Yes	Yes	No	No	No
Bernard et al.[17]	2018	Yes	Yes	No	Yes	No	No	No
Bousson et al.[26]	1999	Yes	Yes	Yes	Yes	No	No	No
Bret et al.[15]	1994	Yes	Yes	Yes	No	No	No	No
Brunereau et al.[9] (1)	1996	NA	NA	NA	NA	NA	NA	NA
Brunereau et al.[9] (2)	1996	NA	NA	NA	NA	NA	NA	NA
Brunereau et al.[9] (3)	1996	NA	NA	NA	NA	NA	NA	NA
Brunereau et al.[9] (4)	1996	NA	NA	NA	NA	NA	NA	NA
Brunereau et al.[9] (5)	1996	NA	NA	NA	NA	NA	NA	NA
Brunereau et al.[9] (6)	1996	NA	NA	NA	NA	NA	NA	NA
Chen CJ et al.[27] (1)	1998	Yes	Yes	Yes	Yes	No	No	No
Chen CJ et al.[27] (2)	1998	No	No	Yes	Yes	No	No	No
Chen PM et al.[28]	2018	No	Yes	Yes	Yes	No	No	No
Chen PY et al.[29]	2019	No	Yes	Yes	Yes	No	No	No
Chng et al.[30]	2004	No	No	Yes	No	No	No	No
Clayton et al.[31]	2020	Yes	Yes	Yes	No	No	No	No
Copelan et al.[20] (1)	2018	No	Yes	Yes	Yes	No	No	No
Copelan et al.[20] (2)	2018	No	Yes	Yes	Yes	No	No	No
Copelan et al.[20] (3)	2018	No	Yes	Yes	Yes	No	No	No
Copelan et al.[20] (4)	2018	No	Yes	Yes	Yes	No	No	No
Deopujari et al.[32]	1995	Yes	Yes	Yes	No	No	No	No
El Asri et al.[10]	2013	Yes	Yes	Yes	No	No	No	No
Enokizono et al.[22] (1)	2017	Yes	Yes	Yes	No	No	No	Yes
Enokizono et al.[22](2)	2017	Yes	Yes	Yes	No	No	No	Yes
Ernst et al.[33] (1)	1997	No	Yes	Yes	No	No	No	No
Ernst et al.[33] (2)	1997	Yes	Yes	Yes	No	No	No	No
Ernst et al.[33] (3)	1997	Yes	No	No	No	No	No	No
Foreman et al.[34]	2013	No	No	Yes	No	No	No	No
Gaensler et al.[35]	1989	NA	NA	NA	NA	NA	NA	NA
Gobin et al.[36] (1)	1992	NA	NA	NA	NA	NA	NA	NA
Gobin et al.[36] (2)	1992	NA	NA	NA	NA	NA	NA	NA
Gobin et al.[36] (3)	1992	NA	NA	NA	NA	NA	NA	NA
Gobin et al.[36] (4)	1992	No	No	Yes	No	No	No	No
Gobin et al.[36] (5)	1992	No	No	Yes	No	No	No	No
Gross et al.[37] (1)	2014	No	Yes	Yes	No	No	No	No
Gross et al.[37] (2)	2014	Yes	Yes	Yes	No	No	No	No
Hähnel et al.[38]	1998	Yes	Yes	Yes	Yes	No	No	No
Haryu et al.[39]	2014	Yes	Yes	Yes	No	No	No	No
Iwase et al.[40]	2020	Yes	Yes	Yes	No	No	No	No
Joseph et al.[41]	2000	No	Yes	Yes	No	No	No	No
Jun Li et al.[18]	2004	No	Yes	Yes	No	No	No	No
Kalamangalam et al.[21]	2002	No	No	Yes	Yes	No	No	No
Kamio et al.[11]	2015	No	Yes	Yes	No	No	No	No
Khan et al.[42]	2009	No	Yes	No	No	No	No	No
Kim HJ et al.[43]	2015	Yes	No	No	Yes	No	No	No
Kim NH et al.[44]	2011	Yes	Yes	No	Yes	No	No	No
Kim WY et al.[45]	2016	Yes	Yes	Yes	No	No	No	No
Kleeberg et al.[46]	2010	No	Yes	Yes	No	No	No	No
Kulwin et al.[47]	2012	No	Yes	Yes	No	No	No	No
Kvint et al.[48]	2020	Yes	No	No	Yes	No	No	No
Lagares et al.[49]	2007	No	Yes	Yes	No	No	No	No
Lv et al.[50]	2011	No	Yes	Yes	No	No	No	No
Mascalchi et al. [51] (1)	1996	Yes	Yes	Yes	No	No	No	No
Mascalchi et al.[51] (2)	1996	Yes	Yes	Yes	No	No	No	No
Narita et al.[52]	1992	No	No	Yes	No	No	No	No
Ogbonnaya et al.[53]	2011	Yes	No	Yes	No	No	No	No
Pannu et al.[54]	2004	No	Yes	Yes	Yes	No	No	No
Partington et al.[55] (1)	1992	NA	NA	NA	NA	NA	NA	NA
Partington et al.[55] (2)	1992	Yes	Yes	No	No	No	No	No
Patsalides et al.[16]	2010	No	Yes	Yes	Yes	No	No	No
Peethambar et al.[16]	2018	Yes	Yes	No	Yes	No	No	No
Peltier et al.[56]	2011	No	Yes	No	Yes	No	No	No
Perkash et al.[57]	2002	Yes	No	Yes	No	No	No	No
Pop et al.[58]	2015	No	Yes	Yes	Yes	No	No	No
Renner et al.[59]	2006	Yes	Yes	Yes	No	No	No	No
Ricolfi et al.[60] (1)	1998	NA	NA	NA	NA	NA	NA	NA
Ricolfi et al.[60] (2)	1998	No	Yes	Yes	No	No	No	No
Ricolfi et al.[60] (3)	1998	Yes	Yes	Yes	No	No	No	No
Ricolfi et al.[60] (4)	1998	Yes	Yes	No	No	No	No	No
Ricolfi et al.[60] (5)	1998	Yes	Yes	Yes	No	No	No	No
Rocca et al.[61]	2019	Yes	Yes	Yes	No	No	No	No
Rodriguez Rubio et al.[62]	2019	No	Yes	Yes	No	No	No	No
Roelz et al.[63]	2015	No	Yes	Yes	Yes	No	No	No
Satoh et al.[64]	2005	No	No	Yes	No	Yes	Yes	No
Shimizu et al.[65]	2019	No	Yes	No	No	No	No	No
Singh et al.[66]	2013	Yes	Yes	Yes	No	No	No	No
Sorenson et al.[67]	2019	Yes	Yes	Yes	No	No	No	No
Sugiura et al.[68]	2009	No	Yes	Yes	Yes	No	No	No
Sun et al.[69]	2019	Yes	Yes	Yes	No	No	No	No
Tanaka et al.[70]	2017	Yes	Yes	No	No	No	No	No
Tanoue et al.[71]	2005	Yes	Yes	Yes	No	No	No	No
Trop et al.[72]	1998	Yes	Yes	Yes	No	No	No	No
Tsutsumi et al.[73]	2008	Yes	Yes	No	Yes	No	No	No
Van Rooij et al.[74] (1)	2007	Yes	Yes	Yes	No	No	No	No
Van Rooij et al.[74] (2)	2007	Yes	Yes	Yes	No	No	No	No
Van Rooij et al.[74] (3)	2007	No	Yes	Yes	No	No	No	No
Versari et al.[75] (1)	1993	Yes	Yes	No	Yes	No	No	No
Versari et al.[75] (2)	1993	Yes	No	No	No	No	No	No
Wang et al.[76]	2019	No	Yes	Yes	No	No	No	No
Wiesmann et al.[14]	2000	No	Yes	Yes	No	No	No	No
Willinsky et al.[77]	1990	No	No	Yes	No	No	No	No
Wrobel et al.[78] (1)	1988	Yes	Yes	No	No	No	No	No
Wrobel et al.[78] (2)	1988	NA	NA	NA	NA	NA	NA	NA
Wrobel et al.[78] (3)	1988	No	No	Yes	No	No	No	No
Yoshida et al.[79]	1999	No	Yes	Yes	No	No	No	No
Zhang et al.[80]	2018	Yes	No	Yes	Yes	No	No	No

*NA*, not available

### Associations between sociodemographic, clinical and MRI variables, and CVF’s onset and outcomes

We initially investigated the association between age, gender, and outcome among the 100 patients with CVFs through non-parametric correlations and crosstabs, respectively. In both cases, results were not significant, suggesting that outcome was not related to the age (*r* = 0.065, *p* = 0.56) or gender (*χ*^2^ = 3.163, *p* = 0.075). We then tested for an association between an initial misdiagnosis and the disease’s outcome, but the chi square test was not significant (*χ*^2^ = 0.194, *p* = 0.66), suggesting that those who had an initial misdiagnosis had similar outcomes compared to those whose CVFs were diagnosed correctly at symptoms’ onset.

As for the association between diagnostic delay (in months) and type of onset, a non-parametric ANOVA (Kruskal–Wallis test (2) = 15.540, *p* < 0.001) evidenced that those with an acute onset had a significantly lower interval to diagnosis compared to those with a progressive (*p* < 0.001) or multiphasic one (*p* = 0.049). All other comparisons were not significant. Interestingly, the association between diagnostic delay and outcome was also significant (*U* = 432.000, *z* =  − 1.960, *p* = 0.050), with patients who experienced a disability or exited receiving their diagnosis months later compared to patients who experienced a good recovery.

As for the association between the presence of specific symptoms at onset (e.g., ataxia, sphincteric disturbances, motor or sensory ones) and diagnostic delay, those who experienced sensory symptoms at onset received their diagnosis of CVFs later than those who did not experience them (*U* = 749.000, *z* = 2.247, *p* = 0.025), while all other comparisons were not significant. As a follow-up analysis, and to better understand the unique contribution of sensory symptoms in explaining the diagnostic delay, we tested for the presence of significant differences in diagnostic delay between those who experienced only sensory symptoms at onset (*N* = 8) and those who experienced sensory symptoms together with other ones (*N* = 16). Though the Mann–Whitney *U* test is non-significant (*U* = 75.500, *z* = 1.308, *p* = 0.20), the between-groups effect size was medium (Hedge’s *g* = 0.65), suggesting that—with a larger sample size—this comparison would have reached the significance. As for the association between the presence of specific symptoms at onset and outcome, none of the chi square tests reached the significance.

Finally, we examined the association between spinal MRI findings at diagnosis, diagnostic delay, and outcome. In these analyses we focused exclusively on MRI swelling, T2 hyperintensities, flow voids, and contrast enhancement due to extremely low incidence of other MRI findings (DWI, T2* abnormalities, and thrombosis). Results showed that MRI findings were unrelated with both diagnostic delay and CVF outcome.

Detailed results, including frequencies, percentages, means, and standard deviations, separately for each group, are reported in the Supplementary Materials.

## Discussion

The diagnosis of CVFs is challenging and often requires the expertise of highly specialized centers, leading to potential delays in diagnosis that can impact clinical outcomes and patients’ quality of life. Many patients, including the case discussed, receive a correct diagnosis only months or years after the onset of symptoms, when irreversible damage may have already occurred. One of the major challenges in diagnosing CVFs is that they are rarely considered in the initial diagnostic workup of myelopathies. Our CVF case was a starting point to conduct an analysis on the possible way to improve the outcome and to look for reliable clinical or radiological signs that could aid an earlier diagnosis.

### Demographic features

Our analysis outlined that the mean age of onset was 56 years old, with most patients being males. These findings are in line with what has been described in a similar review conducted in 2013 [10], confirming CVFs as being a disease mainly affecting middle-aged patients, even though a few pediatric cases have been reported [7].

### Clinical characteristic

The most prevalent type of onset was “progressive,” while the “acute” one was much less represented (13%) compared to the 25% reported in the literature [2], possibly because “multiphasic” onsets were considered “acute” in those other studies. The most common complaints at beginning were motor deficits (either paraparesis or quadriparesis) followed by sensory symptoms, sphincteric disturbances, dizziness, ataxia, and brainstem symptoms (Fig. 5). Interestingly, we found that only 9% of patients had brainstem signs, whereas El Asri and colleagues reported their presence in one-third of the patients [10]; this discrepancy may be due to the different definition of “brainstem signs” between the studies. It was also found that patients presenting with brainstem signs tended to have a shorter time to reach a correct diagnosis (see the “Diagnostic delay” section), which is consistent with current literature [11]. This may be because patients with brainstem signs are often mistaken for having a stroke and are promptly admitted to the emergency room. In cases where the onset is progressive and the pattern is that of an ascending myelopathy (which is the most common pattern), brainstem signs are less likely to appear early, and by the time they do, other symptoms may already be irreversible [2]. Another interesting finding is that patients presenting with only sensory symptoms tended to receive a correct diagnosis much later than those presenting with other symptoms. The most likely explanation is that sensory symptoms are common, easily missed during neurological examination, and their importance is often underestimated by clinicians and by patients themselves. Sensory symptoms are considered less disabling than motor symptoms, so patients may not consult a neurologist until motor symptoms occur, while neurologists may underestimate the subtle onset of sensory findings, often attributing them to radiculopathies or peripheral neuropathies.

### Diagnostic delay

In this study the mean diagnostic delay was 5 months, a result slightly shorter than what had been previously reported (6–12 months) [7, 10]; this minimal difference with studies conducted years ago imply that very few progresses have been made in diagnosing CVFs during the last few decades. Interestingly, our patients with acute symptoms were more likely to be diagnosed correctly and sooner compared to those with progressive or multiphasic onsets. This could be because patients with acute symptoms are more likely to seek medical attention promptly, while those with progressive symptoms may delay seeking medical help for months, as stated in the “Clinical characteristics” section. This concept is of utmost importance since our analysis outlined that diagnostic delay has a significant impact on the outcome (see the “Outcome” section). As a matter of fact, patients experiencing the poorest prognoses (severe disability or death) had the longest time-to-correct diagnosis interval implying a relationship between these two variables. In other words, a longer diagnostic delay was often associated with a worse clinical outcome, suggesting that early diagnosis could not only lead to a reduction in mortality rate but also to a noticeable reduction of the residual disability. Although several studies have drawn the same conclusion in the past, our study managed to statistically support this hypothesis. In contrast, another study by Kamio et al. did not find a correlation between disease duration and prognosis, but did emphasize the importance of prompt and accurate diagnosis for improving symptoms and avoiding poorer outcomes (see the “Outcome” section) [11]. Of note, in the past some authors reported that even paraplegia can be reversible if the fistula is treated before the occurrence of ischemic and gliotic changes, pointing out the importance of early diagnosis and treatment [13, 14].

### Misdiagnosis

In this setting, reaching the correct diagnosis in the shortest possible time and minimizing the misdiagnosis rate is pivotal. According to our numbers, more than half of the patients were initially misdiagnosed as having other diseases, including our own patient. This is a much more discouraging result than the previous 40.2% misdiagnosis rate reported by Kun Hou et al. in their review [6]. The most common reported misdiagnoses were spinal dural A-V fistulas [9], myelitis [15], tumors (mainly lymphoma [16] and glioma [17]), and strokes [13] (see Fig. 4). In one case stroke was suspected twice before the fistula was discovered [18], suggesting that CVF diagnosis is still challenging. Even if in terms of outcome, we did not find any statistically relevant difference between patients who received misdiagnosis and the ones who did not; misdiagnosis could potentially contribute to diagnostic delay, which in turn is associated with poorer outcomes.

It is important to notice that (1) mildly elevated CSF protein and absence of CSF pleocytosis (“albumino-cytological dissociation”) may occur in arteriovenous fistula and therefore should not necessarily be attributed to idiopathic transverse myelitis or Guillain-Barrè syndrome; (2) post steroid worsening should raise the suspicion about a non-inflammatory disease of the spinal cord, particularly a spinal or an intracranial fistula [19].

### Imaging

While conventional angiography is still to be considered the gold standard for definite diagnosis of CVFs, MRI can strongly aid the diagnosis and dramatically shorten the time to diagnosis, especially when MRA sequences or contrast studies are carried out. Abnormal vascular flow voids, which are tortuous and dilated veins generally found on the dorsal or ventral surface of the spinal cord, were eventually found in 81.6% of patients, even when they were not reported initially [20, 21]. A high index of clinical suspicion is then required to carefully evaluate MRI images looking for flow voids so to reduce the interval to diagnosis and, accordingly, achieve a better outcome. Moreover, in the appropriate clinical context, flow voids help distinguishing CVFs from all other mimics (except spinal fistulas). Unfortunately, all other imaging features (i.e., T2/FLAIR hyperintensities and spinal cord swelling) are nonspecific. An interesting description was made by Copelan et al. who reported spinal edema as having a “a tigroid pattern” with geographic central medullary edema and sparing of the periphery as well as internal linear segments [20]. However, they did not include all cases of CVFs, making this differentiation based on tigroid appearance less suitable for generalization. Several other studies tried to find peculiar image findings (i.e., the “black butterfly sign” by Enokizono and colleagues [22]) but these remain isolated observations.

### Outcome

Our analysis did not disclose any relationship between age, sex, and outcome, supporting the current knowledge about CVFs [10] and implying that the prognosis can be severe even in otherwise healthy young subjects. In our study sample, the percentages of moderate and poor recovery/death were 41.3% and 23.8%, respectively, while good recovery was only 32.9% which is consistent with the literature [10] and highlights that CVFs can still result in moderate/severe disability in two-thirds of cases. Moreover, there was no statistical relationship found between the presence of a specific subset of symptoms at onset and the outcome, suggesting that more compromised patients at onset do not necessarily have a worse prognosis. Similar findings have been reported in the literature, particularly regarding the lack of correlation between the severity of symptoms at onset and prognosis, except when signs of brainstem dysfunction are present, possibly due to the involvement of respiratory and cardiovascular centers in the brainstem [10, 11]. Unfortunately, no highly suggestive pattern of CVF symptoms that could shorten the time to diagnosis and lead to a better prognosis was identified in the analysis (see the “Diagnostic delay” section above).

## Limitations

Our review has some intrinsic limitations: (1) it only includes Italian- and English-written articles, excluding some potentially interesting reports written in other languages; (2) it encompasses studies ranging from 1988 to 2021 during which time myelography has been substituted by MRI and MRI itself has become progressively more sophisticated so it was sometimes difficult to compare radiological data among the studies; (3) publications are mostly limited to single case reports and small case series; (4) many patients were lost on follow-up or received a very close range follow-up so that their actual long-term outcome is unknown; (5) in some cases, clinical data were scarce.

## Conclusions and future directions

CVFs are rare and treatable conditions but, since their first clinical description, few progresses have been made in their early diagnosis. Despite the several innovations in surgery and neuroimaging introduced during the last four decades, CVFs still carry a moderate/severe grade of disability in two-third of cases; among the reasons we recognize late diagnosis and treatment. Our analyses show that diagnostic delay is more often associated with worse clinical outcomes, suggesting that early diagnosis could not only lead to a reduction of mortality rate but also to a noticeable reduction in residual disability. Interestingly, the latter is not associated with the severity of clinical picture, so more compromised patients do not necessarily show a worse outcome. Misdiagnosis itself is not associated with a poorer outcome but it can increase diagnostic delay which is, in turn, associated with poorer recoveries.

Unfortunately, we were not able to recognize any highly suggestive (“red flags”) CVF’s pattern of symptoms to shorten the time to diagnosis, but we can empirically suggest considering CVFs and conduct an angiography including cerebral vessels in a patient with slowly progressing/relapsing myelopathy when myelitis routine work-up is inconclusive.

**Fig. 6:**
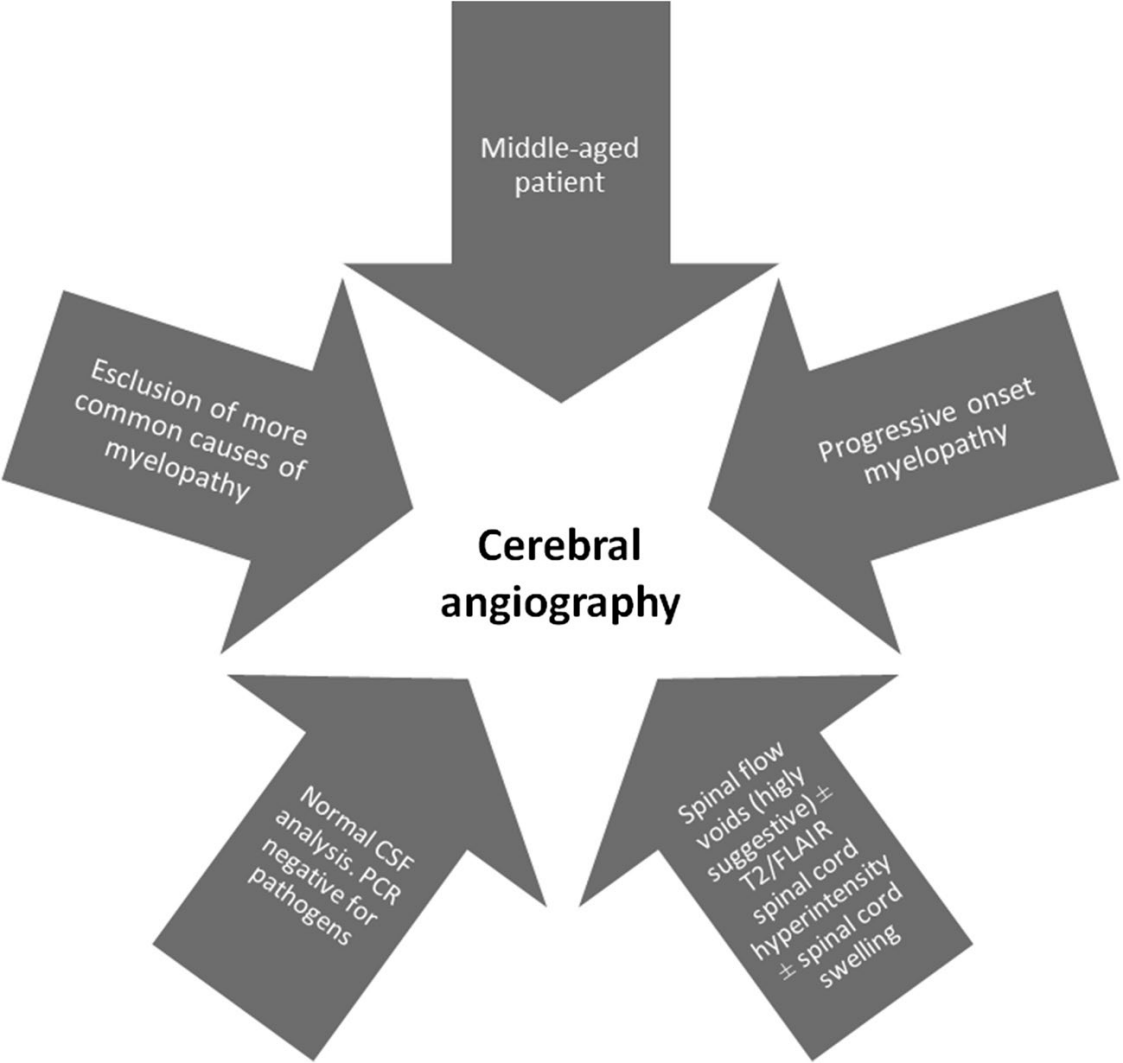
Red flags for performing a cerebral angiography

The findings also emphasize the importance of careful investigation of spinal flow voids in appropriate clinical contexts, as they can provide valuable clues for CVFs and help distinguish them from other mimics, except for spinal fistulas. Prompt extension of angiographic studies to intracranial vessels is suggested when spinal angiography is unremarkable in suspected cases of CVFs [9-11]. Other imaging features were found to be non-specific and could potentially lead to misdiagnosis. Suggestions on when to perform a cerebral angiography are reported in Fig. 6.

In conclusion, a multidisciplinary approach is needed to better understand whether early treatment improves patients’ prognosis and quality of life, and whether a combined clinical-radiological predictive score could help to decide when to perform a cerebral angiography in a patient with otherwise unexplained myelopathy.

## Data Availability

The authors confirm that the data supporting the findings of this study are available within the article and its supplementary materials. Extracted raw data are available on request from the corresponding author, AP.
